# Can we trust computers to assess the cognition of stroke patients? A systematic review

**DOI:** 10.3389/fneur.2023.1180664

**Published:** 2023-05-25

**Authors:** Qi Zhang, Jia-Hang Wei, Xue Fu, Xin Liu, Xin-Yi Li, Wei Liu, Zhong-Liang Liu, Xiao-Qin Duan, Bin Zheng

**Affiliations:** ^1^Department of Rehabilitation Medicine, Jilin University Second Hospital, Changchun, China; ^2^Jilin University, Changchun, China; ^3^Changchun University of Chinese Medicine, Changchun, China; ^4^School of Computer and Communication Engineering, University of Science and Technology Beijing, Beijing, China; ^5^Surgical Simulation Research Lab, Department of Surgery, University of Alberta, Edmonton, AB, Canada

**Keywords:** cognitive assessment screening instrument, stroke, cognitive impairments, computer-aided design, review

## Abstract

**Purpose:**

To summarize the classification of computerized cognitive assessment (CCA) tools for assessing stroke patients, to clarify their benefits and limitations, and to reveal strategies for future studies on CCA tools.

**Methods:**

A literature review was performed using PubMed, Embase, Scopus, JAMA Network, Cochrane Library and PsycINFO databases from January 1st, 2010, to August 1st, 2022. Two authors independently screened the literature following the same criteria, evaluated the study quality, and collected data from the articles.

**Results:**

A total of 8,697 papers were acquired from the six databases. A total of 74 potentially eligible articles were selected for review. Of these, 29 articles were not relevant to this research, 3 were reviews, 2 were not written in English, and 1 was on an ongoing trial. By screening the references of the reviews, 3 additional articles were included in this study. Thus, a total of 42 articles met the criteria for the review. In terms of the CCA tools analyzed in these studies, they included five types: virtual reality (VR)-based, robot-based, telephone-based, smartphone-based, and computer-based cognitive assessments. Patients' stages of the disease ranged from the subacute phase and rehabilitation phase to the community phase. A total of 27 studies supported the effectiveness of CCA tools, while 22 out of 42 articles mentioned their benefits and 32 revealed areas for future improvement of CCA tools.

**Conclusions:**

Although the use of CCA tools for assessing the cognition of post-stroke patients is becoming popular, there are still some limitations and challenges of using such tools in stroke survivors. More evidence is thus needed to verify the value and specific role of these tools in assessing the cognitive impairment of stroke patients.

## 1. Introduction

Stroke is now the leading cause of death and disability globally (1, 2). Post-stroke cognitive impairment (PSCI) is one of the most common complications of stroke. It can impair the patients' cognition in terms of their attention, executive function, memory, language, and visuospatial function, among others. Additionally, PSCI aggravates patients' movement disorders, affects the progress of their rehabilitation, and increases the rates of disability and mortality (3). The incidence of PSCI is as high as 80.97% among those who suffer a stroke (4). If PSCI cannot be effectively controlled, it can adversely affect mental functioning, and even lead to rapidly progressive dementia. In other words, PSCI places a severe burden on families and society.

PSCI is defined as a series of syndromes that meet the diagnostic criteria for cognitive impairment within 6 months after the clinical event of a stroke. In clinical practice, several popular neuropsychological tests have been used to assess cognitive function in association with PSCI, including Mini-Mental State Examination (MMSE), Montreal Cognitive Assessment Scale (MoCA), and the National Institute of Neurological Disorders and Stroke and the Canadian Stroke Network (NINDS-CSN) standardized test (5, 6). However, these traditional neuropsychological scales/tests are associated with certain limitations, such as being time-consuming and subjective (7). In recent years, the popular methods of cognitive assessment have gradually shifted from paper-and-pencil testing to computerized assessment, involving the use of computers, digital tablets, handheld devices, or other digital interfaces to collect data, scores or evaluate neurological dysfunctions. Sternin reported their 35-year experience of using computerized cognitive assessment (CCA). They claimed that CCA can save time, increase scoring accuracy, and be applied to large populations in a robust and efficient manner (8). However, to the best of our knowledge, there has been no systematic evaluation of CCA tools describing their advantages over conventional paper-and-pencil-based assessment.

Against this background, we conducted a systematic review to determine the benefits of using CCA tools in stroke survivors. We also intended to determine the challenges associated with applying such tools and how we can enhance our assessment strategies in stroke management. Specially, in this study, we aim to answer the following questions raised in previous reports: (1) How many types of CCA tools are available? (2) What are the benefits of using CCA tools? (3) What are the limitations of CCA tools? (4) How can CCA tools be improved for future applications?

## 2. Methods

### 2.1. Eligibility criteria

The criteria for the inclusion of studies in this research were as follows:

- Population: stroke patients with impairment of cognition or cognitive domains.
- Intervention: cognitive assessment with computerized tools.
- Comparison/analyses used: no specific requirements.
- Outcomes: cognitive domains involved, patient compliance, specificity and sensitivity, pros and cons of assessment, side effects, use of care services.
- Study type: clinical trials, analytical studies, primary research, studies on individuals with a clinical diagnosis of cognitive impairment after stroke.
- Publication date and language: limited to articles published from January 1st, 2010, to August 1st, 2022, in English.

Studies were excluded based on the following criteria:

- Study type: case reports, conferences, expert consensus, animal studies, abstracts, guidelines, comments, reviews that included a range of study designs or conditions unless they provided separate data for clinical trials with stroke survivors.
- Language: report written in a language other than English.

### 2.2. Information sources and search strategies

The publication data, study type and language filters corresponding to the eligibility criteria were used. Additionally, the following terms were included in different combinations (Supplementary Table S1): stroke, cerebral hemorrhage, hemorrhage, brain infarction, infarction, cerebral infarction; and cognition, metacognition, cognition disorders; and evaluation, assessment, mental status and dementia tests, neuropsychological tests; and compute^*^, intelligent^*^. These search terms were entered as any field (title, mesh, keyword, abstract, main text) in PubMed, Embase, Scopus, JAMA Network, Cochrane Library and PsycINFO for searches of reports published from January 1st, 2010, to August 1st, 2022. The complete search strategies for each database can be found in the Supplementary Table S1.

### 2.3. Study selection and data extraction

Two authors (QZ and JW) independently selected abstracts to retrieve according to the inclusion and exclusion criteria. If the eligibility criteria were met, the full text was obtained and read for further selection. Data were independently extracted by two authors (QZ and JW) using a predetermined data collection template that contained title, authors, institution, study design, year of publication, subjects, groups, stage of stroke, intervention name, involved cognitive domains, purpose of intervention, basic architecture, main characteristics, other assessment, advantages, disadvantages, PSCI diagnosis, outcomes, conclusion, and basic mechanism. In addition, a subsequent cross-check was performed to ensure the accuracy of study selection and data extraction. Inconsistencies were resolved through discussion or by asking the corresponding author for advice until a consensus was reached.

### 2.4. Assessment of risk of bias

Two authors (QZ and XF) independently assessed the risk of bias among the included studies by using the modified Jadad scale (9) for included randomized controlled trials, and using the Newcastle-Ottawa Scale (NOS) (10) for included case-control studies and cohort studies. They also examined cross-sectional studies using the 11-item checklist recommended by the Agency for Healthcare Research and Quality (AHRQ) (11). The above tools that were adapted for use in this study can be found in the Supplementary Methods 1–4. Based on these tools, the overall rating of the general methodological quality of each study would be reported as high, moderate, or low. To allow comparison of the study quality across different study types, a summary score of “low” (Jadad 1–3, NOS 0–4, AHRQ 0–3), “moderate” (NOS 5–6, AHRQ 4–7), or “high” (Jadad 4–7, NOS 7–9, AHRQ 8–11) was assigned. Concordant agreements were achieved through discussion by two researchers. If disagreements occurred, the corresponding author would be asked for advice to reach agreement.

## 3. Results

### 3.1. Study selection

**Figure 1:**
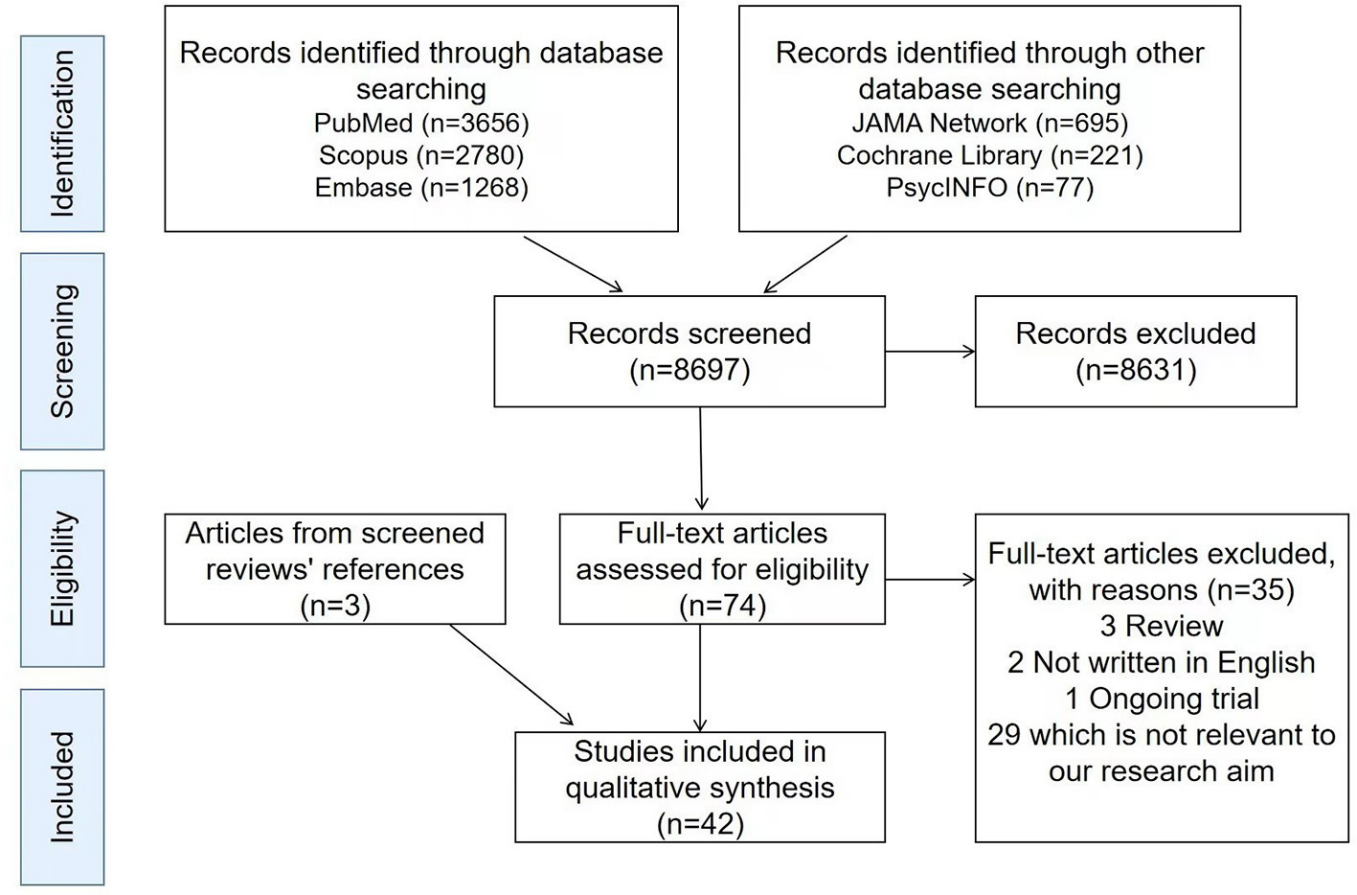
Reference screening flow chart. A total of 8,697 titles and abstracts were acquired from six databases: 3,656 from PubMed, 2,780 from Scopus, 1,268 from Embase, 695 from JAMA Network, 221 from Cochrane Library, and 77 from PsycINFO. The remaining 8,697 titles, abstracts, and methods were initially screened, of which 74 potentially eligible articles were selected for further review. Of these 74 articles, 29 articles were not relevant to our research aim or we were unable to extract relevant information, three articles were reviews, two articles were not written in English, and one was on an ongoing trial. Besides, by screening the references of the identified reviews, three additional articles were included in our study. A total of 42 full-text articles met the eligibility criteria for this systematic review.

A total of 8,697 titles and abstracts were acquired from six databases: 3,656 from PubMed, 2,780 from Scopus, 1,268 from Embase, 695 from JAMA Network, 221 from Cochrane Library, and 77 from PsycINFO. The remaining 8,697 titles, abstracts, and methods were initially screened, of which 74 potentially eligible articles were selected for further review. Of these 74 articles, 29 articles were not relevant to our research aim or we were unable to extract relevant information, 3 articles were reviews, 2 articles were not written in English, and 1 was on an ongoing trial. Besides, by screening the references of the identified reviews, 3 additional articles were included in our study. A total of 42 full-text articles (12–53) met the eligibility criteria for this systematic review (Figure 1).

### 3.2. Risk of bias among the included studies

The modified Jadad scale was used to assess the risk of bias for 2 randomized controlled trials (Supplementary Table S2), while NOS was used for 2 case-control studies and 18 cohort studies (Supplementary Table S3). In addition, the risk of bias of 20 cross-sectional studies were assessed by AHRQ (Supplementary Table S3). Of the 42 studies in this review, 10 articles were of high quality, 27 articles were classed as of moderate quality, and the remaining 5 articles were of low quality.

### 3.3. Study characteristics and types of CCA tools

Stages of stroke can usually be divided into three phases: subacute phase (within 2 weeks), rehabilitation phase (2–8 weeks), and community phase (8 weeks or more). Of the 42 identified studies, four focused on stroke patients in the subacute phase, 20 studies involved those in the community phase, two targeted patients in the subacute and rehabilitation phase, five involved those in the rehabilitation phase and community phase, and six included stroke patients in all stages, while the phases of the stroke patients in five studies were unclear (Table 1).

**Table 1:** Typical characteristics of computerized cognitive assessment tools in articles.

**Category**	**Assessment tool**	**Authors**	**Origin**	**Year**	**Cognitive domains**	**Stage of stroke**	**CI diagnosised by**	**Verification way**
VR-based	SeeMe virtual interactive shopping environment ^a^	Nir-Hadad et al. (12)	Israel	2017	Executive functions	All stages	Clock drawing test	
	the Cognitive Assessment for Aphasia App (C3A) ^a^	Wall (13); Wall et al. (14)	Australia	2017, 2018	Attention, visuospatial skills, visual memory, executive function	All stages	Standard pen-and-paper cognitive tests	+ (16)
	Multitasking in the City Test (MCT) ^a^	Jovanovski et al. (15)	Canada	2012	Visuoperception, visuoconstruction, memory, attention, executive function	Community	Neuropsychological test	+
	VR Wisc-R Mazes ^a^	Carelli et al. (16)	Italy	2011	Visuo-spatial, executive functions	Community	MMSE	
Robot-based	KINARM exoskeleton robotic Evaluation (BKIN Technologies, Canada) ^a^	Singh et al. (17); Mostafavi et al. (18)	USA; Canada	2016, 2017	Spatial planning, working memory, visual processing	Community	Visual cognition assessment (VICA); robotic assessment	(13)
Telephone-based	Modified telephone interview of cognitive status (TICSm) ^b^	Huang et al. (19); Biffi et al. (20); Pendlebury et al. (21)	China; USA; UK	2015, 2017, 2013	Orientation; recent memory, delayed memory; attention, calculation; language	Community	Traditional neuropsychological tests; TICS-m test; modified Petersen criteria, MoCA.	++ (43)
Smartphone-based	Cognitive rehabilitation mobile game (Neuro-World) ^b^	Jung et al. (22)	USA	2019	Orientation, registration, attention, calculation, recall, memory.	NA	MMSE	
	Korean-MMSE with smartphone ^b^	Park et al. (23)	Korea	2017	Orientation, memory, attention, calculation, language, visuospatial	NA	MMSE	
Computer-based	Activity measure for post-acute care (AM-PAC) ^a^	Toglia et al. (24)	USA	2017	Global cognition, cognitive instrumental activities of daily living	Subacute	MoCA	++
	Activity measure for post-acute care (AM-PAC) ^a, b^	Sandel et al. (25)	USA	2013	Applied cognition	All stages	AM-PAC	×
	NeuroTraxTM (known as MindStreams^®^) ^a^	Shopin et al. (26)	Israel	2013	Memory, attention and executive functions	Subacute	MoCA	+
	NeuroTraxTM NeuroTraxTM (known as MindStreams^®^) ^a^	Kliper et al. (27, 28); Boussi-Gross et al. (29)	Israel	2016, 2014, 2015	Global cognition, specific cognitive domains (memory, executive function)	Community	Neurotraxtm	×
	NIH toolbox cognition battery (NIHTB-CB) ^a^	Carlozzi et al. (30); Tulsky et al. (31); Nitsch et al. (32)	USA	2017	Reading, vocabulary, episodic memory, working memory, executive functioning, and processing speed	Community	NIHTB-CB, traditional neuropsychological tests, MMSE	+ (24, 49) (32)
	Computerized CogState battery (CogState Ltd, Australia) ^a^	Cumming et al. (33, 34)	Australia	2012, 2014	Global cognition, particularly attention and visuospatial ability	Subacute	MoCA; neuropsychological battery, quality of life	+ (25, 45)
	Seoul computerized neuropsychological test (SCNT, Maxmedica Inc) ^a^	Kim et al. (35); Yun et al. (36); Kim et al. (37)	Korea	2010, 2015, 2014	Attention, working memory, verbal memory, executive functioning, visuomotor coordination	Rehabilitation, community	MMSE	(52)
	Computerized shape cancellation task ^a^	Ten Brink et al. (38, 39)	the Netherlands	2016	Attention, visual search	Subacute, rehabilitation	MMSE	(53)
	Computerized touchscreen cancellation, sustained attention and spatial working memory ^a^	Dalmaijer et al. (40)	UK	2018	Spatial neglect, attention, working memory	Rehabilitation, community	Touchscreen cancellation, sustained attention, spatial working memory	×
	Computerized Attentional Performance (TAP Mobility), Delis-Kaplan Executive Function System (D-KEFS), Tower of London (TOL-F) ^a^	Schumacher et al. (41)	UK	2019	Attention, executive functions, visuospatial planning	All stages	Neuropsychological tests	(17)
	Ryokansan touch screening test (Ohtsu Computer Corp, Japan) ^a^	Deguchi et al. (42)	Japan	2013	Judgment, processing and discrimination ability, remote and recent memory	NA	MMSE	(14)
	Intelligent cognitive assessment system (ICAS) ^a^	Yip and Man (43)	China	2010	Working memory, orientation to time, semantic memory, calculation, visual recognition, abstract thinking, visual interference, attention span, executive function	All stages	MMSE	++
	Remote acquisition of neuropsychological data (RAND) ^a^	Durisko et al. (44)	USA	2016	Verbal memory, language	NA	RAND system	×
	Computerized visual search task ^a^	Schendel et al. (45)	USA	2016	Attention	Community	Raven's colored progressive matrices, conventional behavioral inattention test (BITC), computerized visual search task	+
	Computerized visual searching task (CVST) ^a^	Van Tuijl et al. (46)	The Netherlands	2020	Information processing	Community	MMSE, CVST	
	PC tests Amunet (NeuroScios GmbH, Austria) ^a^	Wu et al. (47)	China	2016	Spatial navigation	Community	MMSE, MOCA	
	Computerized Wisconsin card sorting test ^a^	Fernández-Andújar et al. (48)	Spain	2014	Executive functioning	Community	Neuropsychological tests	×
	Computerized Iowa Gambling task (IGT) ^a^	Escartin et al. (29)	Spain	2012	Decision-making	Community	IGT, Wechsler adult intelligence scale-III (WAIS-III), word fluency test, Wisconsin card sorting test	+
	Tablet-based symbol digit modalities test (T-SDMT) ^a^	Tung et al. (50)	Taiwan	2016	Information processing	Community	T-SDMT, symbol digit modality test (SDMT)	+
	Computer adaptive testing in Neuro-QOL ^a^	Naidech et al. (51)	USA	2015	Executive function	Rehabilitation, community	QOL assessment	+
	Auditory test of variables of attention (TOVA) ^a^	Wallmark et al. (52)	Sweden	2015	Concentration/ attention	Community	TOVA, Montgomery Åsberg depression rating scale, MOCA	+
	Computer-based visuomotor task (CbVM) ^a^	Tippett et al. (53)	Canada	2013	Visually-guided motor performance	NA	MMSE	×

Of the 42 identified studies, four focused on stroke patients in the subacute phase, 20 studies involved those in the community phase, two targeted patients in the subacute and rehabilitation phase, five involved those in the rehabilitation phase and community phase, and six included stroke patients in all stages, while the phases of the stroke patients in five studies were unclear. Abbreviations: VR, virtual reality; MMSE, Mini-Mental state examination; MoCA, Montreal cognitive assessment; CI, Cognitive impairment; QOL, Quality of life. ^a^in-person using; ^b^remote using. Verified by comparing with control group and can identified CI; Verified by comparing with standard neuropsychological tests; Verified by sensitivity and specificity analysis; × No verifications.

According to the 2021 Chinese Expert Consensus on Post-Stroke Cognitive Impairment Management, the diagnosis of PSCI (no dementia) must be based on baseline cognitive decline and impairment of at least one cognitive domain, while instrumental activities of daily living can be normal or slightly impaired. In all 42 selected studies, assessments for reaching a diagnosis of cognitive impairment (CI) only involved MMSE, MoCA, or other neuropsychological tests or evaluation of quality of life (Table 1), which is not comprehensive.

The CCA tools reported in the 42 studies could be divided into five types: those involving virtual reality (VR)-based, robot-based, telephone-based, smartphone-based and computer-based cognitive assessments (Table 1).

#### 3.3.1. VR-based cognitive assessment tools

Five studies reported the use of VR-based cognitive assessment tools performed in-person, including SeeMe Virtual Interactive Shopping environment (12), Cognitive Assessment for Aphasia App (C3A) (13, 14), Multitasking in the City Test (MCT) (15), and VR Wisc-R Mazes (16). Almost all of these tools focused on stroke patients involved in acute-setting inpatient rehabilitation and community-dwelling patients. They involved the evaluation of executive functions, attention, memory, and visuospatial skills (Table 1). In addition, Nir-Hadad et al. (12) and Carelli et al. (16) verified that the SeeMe Virtual Interactive Shopping environment and VR Wisc-R Mazes could be used to assess executive functions and visuospatial abilities in the daily activities of stroke patients, by comparing them with healthy participants. Meanwhile, Wall (13) and Jovanovski et al. (15) demonstrated the validity of C3A and MCT by comparing with the results obtained for normal subjects, as well as by comparing these approaches with standard pen-and-paper tests (Table 1).

#### 3.3.2. Robot-based cognitive assessment tools

Two studies described the use of the robot-based cognitive assessment tool named KINARM Exoskeleton Robotic Evaluation (BKIN Technologies, Kingston, ON, Canada) performed in person (17, 18). Robotic technologies can provide neuropsychological tasks for assessing visuomotor and cognitive functions (e.g., spatial planning, working memory, visual processing) of stroke survivors living in the community (Table 1). Singh et al. (17) confirmed that KINARM Exoskeleton Robotic Evaluation was an effective computational model for examining visual search, by comparing the results obtained for stroke patients and those obtained for young and old healthy adults (Table 1).

#### 3.3.3. Telephone-based cognitive assessment tools

Properly speaking, telephone-based assessment does not fall within the scope of computerized assessment. But we found that in three studies on remote cognitive assessment of stroke patients in the community, the modified telephone interview of cognitive status (TICSm) was used. Therefore, we did not omit it. The involved cognitive domains in TICSm were orientation, recent memory, delayed memory, attention, calculation, and language (19–21) (Table 1). Of these studies, Pendlebury et al. (21) proved that TICSm is a feasible and valid telephone-based method of testing stroke patients, not only by comparing such patients with a control group and compairing this approach with a standard neuropsychological test, but also by analyzing the sensitivity and specificity of TICSm (21) (Table 1).

#### 3.3.4. Smartphone-based cognitive assessment tools

A cognitive rehabilitation mobile game named Neuro-World and the Korean Version of the MMSE using a smartphone were shown to be appropriate smartphone-based tools for detecting multi-domain impairment of stroke patients (22, 23). It was found that they can be used to assess cognitive functions including orientation, registration, attention, calculation, memory, language, and visuospatial abilities. Both of these approaches were confirmed to be useful given their good agreement with the results of standard assessments (22, 23) (Table 1).

#### 3.3.5. Computer-based cognitive assessment tools

A total of 30 studies on the computer-based cognitive assessment of stroke patients were identified. Of these, 21 studies on cognitive assessment tools focusing on evaluating multi-domain or global cognition featured the following tools: Computerized Activity Measure for Post-Acute Care (AM-PAC) (24, 25), NeuroTrax™ (also known as MindStreams^®^) (26–29), NIH Toolbox Cognition Battery (NIHTB-CB) (30–32), CogState Battery (CogState Ltd., Australia) (33, 34), Seoul Computerized Neuropsychological Test (SCNT, Maxmedica Inc.) (35–37), computerized shape cancellation task (38, 39), computerized touchscreen cancellation, sustained attention and spatial working memory (40), computerized test of Attention Performance, Delis-Kaplan Executive Function System and Tower of London (41), Ryokansan touch panel-type screening test (42), Intelligent Cognitive Assessment System (ICAS) (43), and remote acquisition of neuropsychological data (RAND) (44). Meanwhile, nine studies reported tools for the assessment of cognition for a single domain, including computerized visual search task (45, 46), the PC test Amunet (NeuroScios GmbH, Austria) (47), computerized Wisconsin Card Sorting Test (48), computerized Iowa Gambling Task (IGT) (49), Tablet-based Symbol Digit Modalities Test (T-SDMT) (50), computer adaptive testing in Neuro-QOL (51), auditory test of variables of attention (TOVA) (52), and computer-based visuomotor task (53) (Table 1).

The finding showed that AM-PAC (24) and ICAS (43) were valid tools for screening the cognition of stroke patients, not only by comparing such patients with a control group and comparing these tools with a standard neuropsychological test, but also by analyzing these tools' sensitivity and specificity. Besides, NeuroTrax™ (26), NIH Toolbox (30, 32), computerized CogState Battery (33, 34), computerized visual search task (45), computerized IGT (49), T-SDMT (50), and TOVA (52) were confirmed to be reliable cognitive assessment tools by comparing their results for stroke patients with those for a control group and by comparing these approaches with a standard neuropsychological test. Meanwhile, computer adaptive testing in Neuro-QOL (51) was analyzed by comparing the results for stroke patients with those for a control group and by evaluating the sensitivity/ specificity of this approach. The validity of the remaining computer-based assessment tools was confirmed by comparing stroke patients with a control group (31, 37, 39, 41, 42, 46, 47) (Table 1).

Overall, although only three studies supplied evidence by performing comparisons with a control group/standard neuropsychological test, and by performing sensitivity/specificity analyses, a total of 27 studies (64.3%) had supported the effectiveness of CCA tools.

### 3.4. Benefits and limitations of CCA tools

Of the 42 included articles, 22 mentioned the benefits of CCA tools. As shown in Table 2, 31.8% of the articles clarified that CCA helped clinicians achieve remote cognitive assessment of patients; 27.3% demonstrated that CCA tools were easy for physicians and patients to use; and 18.2% asserted that CCA provided a dynamic assessment of cognitive function, minimized dependence on language skills (aphasia-friendly), and could be used in other clinical populations such as those suffering from Parkinson's disease and Alzheimer's disease. The advantages reported in 13.6% of the articles included reducing the assessment time, increasing scoring accuracy, simulating diverse life situations, requiring no special training, being more feasible than pen-and-paper tests, and a high level of participants' satisfaction. Besides, 9.1% of studies reported that CCA could be programmed with different languages and used at home. Some studies also clarified that CCA could be applied to large populations and implemented in institutions lacking expensive equipment (23). Besides, robot-based CCA could provide support for patients' limbs to reduce fatigue (18). Meanwhile, Durisko et al. (44) emphasized the feasibility of using RAND system for virtual home-based assessment without prior face-to-face contact between a participant and researcher. Wallmark et al. (52) suggested that the auditory versions of such tools could avoid the possible risk of epileptic seizures in patients exposed to flashing screens.

**Table 2:** Benefits and limitations of computerized cognitive assessment (CCA) tools in articles.

**Benefits of CCA**	**Percentage**	**Benefits of CCA**	**Percentage**	**Limitations of CCA**	**Percentage**
Remote assessment (19–23, 25, 44)	7/22 (31.8%)	More feasible than standard pen-and-paper tests (13, 14, 26)	3/22 (13.6%)	Excluded severe language, cognitive, and functional deficits (21–24, 26, 28, 33, 34, 50)	9/15 (60%)
Easy to be used and learned (15, 18, 33, 42–44)	6/22 (27.3%)	High satisfaction (13, 14, 44)	3/22 (13.6%)	Influenced by previous experience with computers or tablets use (14, 16, 50)	3/15 (20%)
Providing a dynamic assessment of function (12, 22, 25, 53)	4/22 (18.2%)	Can be programmed to other languages (26, 43)	2/22 (9.1%)	Difficulty in getting orientation (12, 16)	2/15 (13%)
Aphasia-friendly (13, 14, 41, 43)	4/22 (18.2%)	at-home usability (22, 44)	2/22 (9.1%)	Discomforts (short-term eye strain, fatigue) (12, 14)	2/15 (13%)
Could be used in other clinical populations (17, 31, 43, 51)	4/22 (18.2%)	Applied to large population (25)	1/22 (4.5%)	Not involving detailed manual-handling tasks (42)	1/15 (6.7%)
Reducing assessment time (18, 25, 43)	3/22 (13.6%)	Security (44)	1/22 (4.5%)	Influenced by instrumental, technological and cultural factors in clinical environments (25)	1/15 (6.7%)
Increasing scoring accuracy (26, 43, 53)	3/22 (13.6%)	Auditory version avoided a possible risk for epileptic seizures compared to flashing screens (52)	1/22 (4.5%)	Remote assessment has some possibilities of errors when compared with the conventional in-person assessment (23)	1/15 (6.7%)
Simulating diverse life situations (12, 14, 15)	3/22 (13.6%)	Be implemented in centers having no expensive equipment (23)	1/22 (4.5%)	Failing to react to correct stimuli (52)	1/15 (6.7%)
Requiring no special training or expertise (15, 42, 44)	3/22 (13.6%)	Providing weight support to reduce possible fatigue (18)	1/22 (4.5%)	Missing data (14)	1/15 (6.7%)

Of the 42 included articles, 22 mentioned the benefits of CCA tools. 31.8% of the articles clarified that CCA helped clinicians achieve remote cognitive assessment of patients; 27.3% demonstrated that CCA tools were easy for physicians and patients to use; and 18.2% asserted that CCA provided a dynamic assessment of cognitive function, minimized dependence on language skills (aphasia-friendly), and could be used in other clinical populations such as those suffering from Parkinson's disease and Alzheimer's disease. The advantages reported in 13.6% of the articles included reducing the assessment time, increasing scoring accuracy, simulating diverse life situations, requiring no special training, being more feasible than pen-and-paper tests, and a high level of participants' satisfaction. Among the 42 identified studies, 15 analyzed the limitations of CCA tools. 9 out of these 15 studies (60%) excluded patients with severe language, cognitive, and functional deficits. Moreover, 20% of the articles on these studies reported that the results of CCA were influenced by previous experience using a computer, and patients easily made mistakes when they had rarely used a computer or tablet before the test. Additionally, 13% of the articles described patients becoming disorientated, including suffering discomfort such as short-term eye strain, or fatigue.

Among the 42 identified studies, 15 analyzed the limitations of CCA tools. As shown in Table 2, 9 out of these 15 studies (60%) excluded patients with severe language, cognitive, and functional deficits. Moreover, 20% of the articles on these studies reported that the results of CCA were influenced by previous experience using a computer, and patients easily made mistakes when they had rarely used a computer or tablet before the test. Additionally, 13% of the articles described patients becoming disorientated, including suffering discomfort such as short-term eye strain, or fatigue. Besides, it was shown that, for a novel computerized touch panel-type screening test named Ryokansan et al. (42), the use of the tool did not involve detailed manual-handling tasks. Furthermore, Sandel et al. (25) reported that the implementation of AM-PAC in clinical environments and the success of the project were influenced by instrumental, technological, operational, resource-related, and cultural factors. Park et al. (23) also found that remote assessment was associated with the possibility of errors when compared with conventional in-person assessment, and that special consideration should be made when interpreting scores for attention/calculation and visuospatial functions. Finally, Wallmark et al. (52) and Wall et al. (14) presented certain adverse outcomes in CCA, such as failing to react to correct stimuli and missing data.

### 3.5. Areas for future improvement of CCA studies

A total of 32 studies mentioned areas and strategies for the future development of CCA (Table 3). Overall, 59.4% of studies (19/32) mentioned that further studies should be performed on a larger sample of subjects. In addition, 21.9% of articles (7/32) reported that advanced research should include specific patient groups (e.g., those with severe cognitive impairment or aphasia), while 15.6% (5/32) suggested examining the connection between lesion location and task performance to draw conclusions about the influence of stroke topography. Another five articles (15.6%) advised that the same CCA task should be implemented on various technologies. Besides, 12.5% of articles introduced strategies including the use of more extensive cognitive examinations and the examination of longitudinal test performance to document the typical recovery of function. Moreover, three articles (9.4%) recommended identifying and reducing factors associated with patients' poor performance, such as fatigue, difficulty working under pressure, infection in the acute phase, lack of effort, use of medication, psychiatric history, litigation status, learning disability, hearing loss, employability and quality of life. Three other articles (9.4%) proposed examining acute recovery following stroke onset in further study. Additionally, 6.25% of articles proposed various directions for future work, such as combining CCA with a computational model, investigating the differing clinical demands and resources in various clinical settings, and identifying those with deficits in Cognitive Instrumental Activities of Daily Living (C-IADL) and Domain-specific Health-related Quality of Life (HRQoL) to achieve appropriate intervention, discharge planning, support, and follow-up. Singh et al. (17) also reported that, in future CCA studies, this approach should be combined with eye tracking. Moreover, Nir-Hadad et al. (12) considered examining the ecological validity by comparing performance in the virtual adapted shopping task with performance of a similar task in a supermarket of CCA as a future direction. Van Tuijl et al. (46) also recommended that more etiological research be performed on the specific cognitive consequences of stroke. Carelli et al. (16) reported the need for additional studies on issues relating to human-computer interaction in tests. Finally, Wall et al. (14) suggested exploring clinicians' user acceptance, investigating the different clinical demands and resource implications in varying clinical settings.

**Table 3:** Areas for future improvement of CCA tools.

**Future directions of CCA researches**	**Percentage**	**Future directions of CCA researches**	**Percentage**
Further studies in a larger sample of people (12, 14–16, 21, 22, 24, 33–37, 43, 46–49, 52, 53)	19/32 (59.4%)	Combining with computational model (17, 18)	2/32 (6.25%)
Including specific patient groups (e.g. with severe cognitive impairment or aphasia) (23, 24, 26, 28, 33, 43, 50)	7/32 (21.9%)	Investigating differing clinical demands and resource (14, 39)	2/32 (6.25%)
Examining connection between brain lesion location and task performance (33, 37, 38, 48, 53)	5/32 (15.6%)	Identifying deficits of ability of daily life in stroke patients (24, 51)	2/32 (6.25%)
Examining same task implemented on various technologies (12, 23, 25, 43, 50)	5/32 (15.6%)	Combining with eye tracking (17)	1/32 (3.1%)
Using more extensive cognitive examinations (18, 29, 35, 53)	4/32 (12.5%)	Further examining the ecological validity (12)	1/32 (3.1%)
Examining longitudinal performance to document the typical recovery of cognitive function (25, 30, 35, 47)	4/32 (12.5%)	More etiological research on the specific cognitive consequences (46)	1/32 (3.1%)
Reducing factors of patients' poor performance (19, 30, 52)	3/32 (9.4%)	Human-computer interaction issues in tests (16)	1/32 (3.1%)
Examining in acute phase of stroke (21, 30, 47)	3/32 (9.4%)	Exploring clinicians' user acceptance (14)	1/32 (3.1%)

A total of 32 studies mentioned areas and strategies for the future development of CCA. Overall, 59.4% of studies (19/32) mentioned that further studies should be performed on a larger sample of subjects. In addition, 21.9% of articles (7/32) reported that advanced research should include specific patient groups (e.g., those with severe cognitive impairment or aphasia), while 15.6% (5/32) suggested examining the connection between lesion location and task performance to draw conclusions about the influence of stroke topography. Another five articles (15.6%) advised that the same CCA task should be implemented on various technologies. Besides, 12.5% of articles introduced strategies including the use of more extensive cognitive examinations and the examination of longitudinal test performance to document the typical recovery of function.

## 4. Discussion

This systematic review summarizes the current evidence for the use of CCA tools in post-stroke patients. CCA tools related to stroke in the selected articles were based on VR, robots, telephones, smartphones, and computers. The studies presented here identified these five assessment methods, which would be considered for further research. Each selected study varied in its aim of assessment and measured outcomes, but increasing evidence supports the use of CCA as a clinical or rehabilitation tool. Of the 42 papers included in this study, 27 provided evidence for the effectiveness of CCA tools. Moreover, it was definitively concluded that TICSm, AM-PAC and ICAS are reliable and valid CCA tools for post-stroke patients.

Except for the benefits reported by Sternin et al. (8), 22 out of 42 articles expounded on the advantages of CCA tools. First, remote assessment, rather than in-person assessment, was assessed in seven studies (19–23, 25, 44). Remote CCA makes it possible to communicate with patients at home (22, 44) or elsewhere over the telephone or via an app on a smartphone, without physical contact. This would be critical for stroke patients and physicians in situation like the COVID-19 pandemic. Second, several studies considered the benefits of CCA such as the ease of use (15, 18, 33, 42–44), even aphasia-friendly (13, 14, 41, 43). Compared with traditional pen-and-paper cognitive tests, stroke patients preferred a CCA tool featuring two large external-response keys (“yes” and “no” buttons) (33). In addition, it was shown that touch panel-based screening tests could be easily understood and performed by patients due to the use of clear images and simple methods (42). Third, the CCA tools could be used to dynamically assess cognitive function (12, 22, 25, 53). Taking the AM-PAC assessment sessions as an example, these take only 7.9 min on average for data acquisition, and they can be used to track and assess patients' function in situation ranging from institutional to community settings (25). Besides, CCA tools can be used in other languages (26, 43), different situations (12, 14, 15), and clinical populations (17, 31, 43, 51). They may provide support for patients (18) and be associated with higher satisfaction (13, 14, 44) than standard pen-and-paper tests.

However, 15 out of 42 articles revealed limitations of CCA tools as follows. First, patients with severe language, cognitive, and functional deficits were excluded from the studies on CCA tools. Little evidence on the application of CCA tools in severe stroke patients was thus reported, which is a research gap that should be bridged in further work. Second, the results of CCA may be influenced by previous experience of using a computer (14, 16, 50). There is thus a need for some computer or CCA tool training before inclusion in clinical trials in this field, especially for the elderly. Third, several studies reported that stroke patients became disorientated (12, 16) or even experienced discomfort (12, 14) especially when they were immersed in a complex VR environment. Patients complained of short-term eye strain, fatigue, or slight discomfort in these studies. In addition, in contrast to pen-and-paper tests, CCA tools may suffer certain potential problems during their designing (42), setting (25), installation, and running (14, 23, 52). These factors must be considered in the future development and application of CCA tools.

Furthermore, future directions for studies on CCA tools were reported in 32 articles. The top three suggestions for future studies were to expand the sample size, to include stroke patients with severe functional impairment, and to examine the connection between the stroke lesion and CCA performance. Interestingly, besides future studies strictly within the medical field, some CCA studies with an interdisciplinary status (in the medical and engineering fields) were also proposed, such as those examining the same task on various technologies (12, 23, 25, 43, 50), combining CCA with computational models (17, 18) and eye tracking (17), and resolving issues associated with human-computer interactions during the tests (16). Because cognitive impairment after stroke interferes with the quality of life in stroke victims (54), 2 out of 42 studies recommended identifying deficits in the ability to perform activities of daily living (C-IADL, HRQoL) in stroke patients (24, 51) in order to diagnose PSCI accurately and comprehensively in future research.

Apart from the future developments listed in Table 3, we identified two future challenges for the development of CCA. One is how to design task-specific tools for PSCI patients. As suggested in the 2016 and 2021 Chinese Expert Consensus on Post-Stroke Cognitive Impairment Management (55), attention, executive function, memory, language ability and visuospatial ability are the five core cognitive domains affected by stroke (56). To accurately describe the cognitive status of a stroke patients, all of these five core domains need to be comprehensively assessed using CCA tools (57). However, we found that 10 of 42 articles focused on only one cognitive domain, while the others mainly assessed two or more cognitive domains. If a CCA included a task-specific assessment tool, it would remain an issue of how best to adjust the order or process of using CCA tools for testing different cognitive domains. To the best of our knowledge, to date, no research on this topic has been performed. The second challenge is how to improve the quality of clinical trials to study CCA tools. In this review, less than one-quater of the studies (10/42) were assessed as being of high quality, and only 2 of them involved randomized controlled trials. Thus, in future studies on CCA tools for PSCI patients, there is a need for high-quality clinical trials.

## 5. Conclusions

The studies identified in this review mainly focused on five types of CCA tools: those for VR-based, robot-based, telephone-based, smartphone-based, and computer-based cognitive assessments. Computer-based cognitive assessment tools were the most studied, but presented mixed results in the cognitive assessment of post-stroke patients. Certain limitations and challenges of using CCA tools in stroke survivors remain to be overcome, and more evidence is needed to verify the value and specific role of these tools in assessing the cognitive impairment of patients with stroke.

### 5.1. Limitations

Some limitations of this study should be presented. First, only two randomized controlled trials were included in this study, which affected the level of evidence of this study. Second, no deep data synthesis or statistical analysis was performed. Third, most of the identified studies still used MMSE or MoCA as a standard method to reveal the advantages or disadvantages of CCA tools, so further studies are needed to improve the objectivity and precision.

## Data availability statement

The original contributions presented in the study are included in the article/Supplementary material, further inquiries can be directed to the corresponding authors.

## Author contributions

X-QD and BZ contributed to the study conception and design. Material preparation, paper collection, and analysis were performed by QZ, J-HW, XF, and X-YL. The first draft of the manuscript was written by X-QD and XL. All authors commented on previous versions of the manuscript, read, and approved the final manuscript.
