# Supplementary material for: Can we trust computers to assess the cognition of stroke patients? A systematic review
Source: Front Neurol. 2023 May 25;14:1180664. doi: 10.3389/fneur.2023.1180664 (PMC10248476; doi:10.3389/fneur.2023.1180664)
Supplement: Supplementary file 1 [file Data_Sheet_1.DOCX]

Supplementary Material

**Can we trust the computer to assess cognition for stroke patients?**

**A systematic review**

Qi Zhang, B.S.^1#^; Jiahang Wei, B.S.^2#^; Xue Fu^#^, B.S.^3^;Xin Liu, Ph.D ^4,5^; Xinyi Li, M.M.^1^; Wei Liu, M.M.^1^; Zhong-liang Liu, M.M.^1^; Xiaoqin Duan, M.D., Ph.D1^*^,5 Bin Zheng, M.D., Ph.D^5*^

## ^#^All three authors are first authors, and contributed equally to the manuscript.

***Corresponding Author:**

## Xiaoqin Duan^*^, MD, PhD, Email: [15204309769@163.com](mailto:15204309769@163.com;).

Bin Zheng^*^, MD, PhD, Email: [bin.zheng@ualberta.ca](mailto:bin.zheng@ualberta.ca;).

**Supplementary Materials Content**

**Table S1.** Summary of search strategies and numbers of papers in PubMed, Embase, Scopus, JAMA Network, Cochrane Library and PsycINFO from Jan 1st, 2010 to Aug 1st, 2022

**Table S2.** Quality evaluation for randomized controlled studies (by modified Jadad scale)

**Table S3.** Quality evaluation for Case-control studies and Cohort studies (by NOS)

**Table S4.** Quality evaluation for Cross-sectional studies (by AHRQ)

**Supplementary Methods 1.** Modified Jadad scale for included randomized controlled trials

**Supplementary Methods 2.** Newcastle-Ottawa Scale (NOS) for case-control studies

**Supplementary Methods 3.** Newcastle-Ottawa quality assessment scale guide for cohort studies

**Supplementary Methods 4.** the 11-item checklist which was recommended by Agency for Healthcare Research and Quality (AHRQ) for cross-sectional studies

This supplementary material has been provided by the authors to give readers additional information about their work.

**Table S1.** Summary of search strategies and numbers of papers in PubMed, Embase, Scopus, JAMA Network, Cochrane Library and PsycINFO from Jan 1st, 2010 to Aug 1st, 2022

| Search terms as any field  (mesh, keyword, title, abstract, main text) | Pubmed | Embase | Scopus | JAMA Network | Cochrane Library | PsychINFO |
| --- | --- | --- | --- | --- | --- | --- |
| stroke & cognition & evaluation & Compute* | 129 | 122 | 119 | 39 | 19 | 0 |
| cerebral hemorrhage & cognition & evaluation & Compute* | 7 | 1 | 5 | 11 | 1 | 0 |
| hemorrhage & cognition & evaluation & Compute* | 288 | 37 | 39 | 17 | 3 | 0 |
| brain infarction & cognition & evaluation & Compute* | 12 | 14 | 25 | 13 | 2 | 0 |
| infarction & cognition & evaluation & Compute* | 28 | 32 | 39 | 27 | 4 | 0 |
| cerebral infarction & cognition & evaluation & Compute* | 10 | 3 | 9 | 11 | 0 | 0 |
| stroke & Metacognition & evaluation & Compute* | 0 | 0 | 1 | 0 | 1 | 0 |
| cerebral hemorrhage & Metacognition & evaluation & Compute* | 0 | 0 | 0 | 0 | 0 | 0 |
| hemorrhage & Metacognition & evaluation & Compute* | 0 | 0 | 0 | 0 | 0 | 0 |
| brain infarction & Metacognition & evaluation & Compute* | 0 | 0 | 0 | 0 | 0 | 0 |
| infarction & Metacognition & evaluation & Compute* | 0 | 0 | 0 | 0 | 0 | 0 |
| cerebral infarction & Metacognition & evaluation & Compute* | 0 | 0 | 0 | 0 | 0 | 0 |
| stroke & Cognition Disorders & evaluation & Compute* | 39 | 2 | 34 | 31 | 5 | 0 |
| cerebral hemorrhage & Cognition Disorders & evaluation & Compute* | 3 | 0 | 3 | 7 | 0 | 0 |
| hemorrhage & Cognition Disorders & evaluation & Compute* | 82 | 1 | 17 | 12 | 2 | 0 |
| brain infarction & Cognition Disorders & evaluation & Compute* | 6 | 0 | 12 | 11 | 1 | 0 |
| infarction & Cognition Disorders & evaluation & Compute* | 14 | 0 | 17 | 21 | 3 | 0 |
| cerebral infarction & Cognition Disorders & evaluation & Compute* | 6 | 0 | 6 | 9 | 0 | 0 |
| stroke & cognition & assessment & Compute* | 203 | 358 | 310 | 48 | 59 | 4 |
| cerebral hemorrhage & cognition & assessment & Compute* | 13 | 4 | 15 | 10 | 3 | 0 |
| hemorrhage & cognition &assessment & Compute* | 498 | 95 | 118 | 18 | 10 | 0 |
| brain infarction & cognition & assessment & Compute* | 17 | 43 | 62 | 13 | 10 | 0 |
| infarction & cognition & assessment & Compute* | 36 | 93 | 104 | 30 | 14 | 0 |
| cerebral infarction & cognition & assessment & Compute* | 16 | 15 | 30 | 10 | 1 | 0 |
| stroke & Metacognition & assessment & Compute* | 0 | 2 | 2 | 0 | 1 | 0 |
| cerebral hemorrhage & Metacognition & assessment & Compute* | 0 | 0 | 0 | 0 | 0 | 0 |
| hemorrhage & Metacognition & assessment & Compute* | 0 | 1 | 1 | 0 | 0 | 0 |
| brain infarction & Metacognition & assessment & Compute* | 0 | 0 | 0 | 0 | 0 | 0 |
| infarction & Metacognition & assessment & Compute* | 0 | 0 | 0 | 0 | 0 | 0 |
| cerebral infarction & Metacognition &assessment & Compute* | 0 | 0 | 0 | 0 | 0 | 0 |
| stroke & Cognition Disorders & assessment & Compute* | 72 | 3 | 107 | 38 | 12 | 4 |
| cerebral hemorrhage & Cognition Disorders & assessment & Compute* | 5 | 0 | 7 | 6 | 0 | 0 |
| hemorrhage & Cognition Disorders & assessment & Compute* | 157 | 0 | 39 | 12 | 2 | 0 |
| brain infarction & Cognition Disorders &assessment & Compute* | 9 | 1 | 26 | 11 | 2 | 0 |
| infarction & Cognition Disorders & assessment & Compute* | 18 | 1 | 36 | 21 | 4 | 0 |
| cerebral infarction & Cognition Disorders &assessment & Compute* | 8 | 0 | 14 | 8 | 0 | 0 |
| stroke & cognition &Mental Status and Dementia Tests & Compute* | 9 | 0 | 11 | 11 | 2 | 1 |
| cerebral hemorrhage & cognition &Mental Status and Dementia Tests & Compute* | 1 | 0 | 1 | 3 | 0 | 0 |
| hemorrhage & cognition &Mental Status and Dementia Tests& Compute* | 39 | 0 | 2 | 6 | 1 | 0 |
| brain infarction & cognition &Mental Status and Dementia Tests & Compute* | 2 | 0 | 2 | 7 | 1 | 0 |
| infarction & cognition &Mental Status and Dementia Tests& Compute* | 5 | 0 | 3 | 9 | 1 | 0 |
| cerebral infarction & cognition & Mental Status and Dementia Tests& Compute* | 2 | 0 | 2 | 6 | 0 | 0 |
| stroke & Metacognition &Mental Status and Dementia Tests& Compute* | 0 | 0 | 0 | 0 | 0 | 0 |
| cerebral hemorrhage & Metacognition &Mental Status and Dementia Tests& Compute* | 0 | 0 | 0 | 0 | 0 | 0 |
| hemorrhage & Metacognition &Mental Status and Dementia Tests& Compute* | 0 | 0 | 0 | 0 | 0 | 0 |
| brain infarction & Metacognition &Mental Status and Dementia Tests & Compute* | 0 | 0 | 0 | 0 | 0 | 0 |
| infarction & Metacognition &Mental Status and Dementia Tests& Compute* | 0 | 0 | 0 | 0 | 0 | 0 |
| cerebral infarction & Metacognition &Mental Status and Dementia Tests & Compute* | 0 | 0 | 0 | 0 | 0 | 0 |
| stroke & Cognition Disorders &Mental Status and Dementia Tests & Compute* | 7 | 0 | 7 | 11 | 0 | 1 |
| cerebral hemorrhage & Cognition Disorders & Mental Status and Dementia Tests& Compute* | 1 | 0 | 1 | 3 | 0 | 0 |
| hemorrhage & Cognition Disorders & Mental Status and Dementia Tests& Compute* | 21 | 0 | 1 | 5 | 0 | 0 |
| brain infarction & Cognition Disorders &Mental Status and Dementia Tests& Compute* | 0 | 0 | 1 | 7 | 0 | 0 |
| infarction & Cognition Disorders &Mental Status and Dementia Tests & Compute* | 3 | 0 | 1 | 9 | 0 | 0 |
| cerebral infarction & Cognition Disorders &Mental Status and Dementia Tests& Compute* | 0 | 0 | 1 | 6 | 0 | 0 |
| stroke & cognition &Neuropsychological Tests & Compute* | 123 | 42 | 225 | 12 | 10 | 5 |
| cerebral hemorrhage & cognition &Neuropsychological Tests & Compute* | 8 | 0 | 20 | 3 | 0 | 1 |
| hemorrhage & cognition &Neuropsychological Tests& Compute* | 547 | 2 | 97 | 3 | 0 | 1 |
| brain infarction & cognition &Neuropsychological Tests & Compute* | 16 | 7 | 55 | 8 | 0 | 0 |
| infarction & cognition &Neuropsychological Tests& Compute* | 35 | 11 | 81 | 9 | 0 | 0 |
| cerebral infarction & cognition & Neuropsychological Tests& Compute* | 13 | 1 | 33 | 6 | 0 | 0 |
| stroke & Metacognition &Neuropsychological Tests& Compute* | 0 | 0 | 2 | 0 | 0 | 0 |
| cerebral hemorrhage & Metacognition &Neuropsychological Tests& Compute* | 0 | 0 | 0 | 0 | 0 | 0 |
| hemorrhage & Metacognition &Neuropsychological Tests& Compute* | 2 | 0 | 0 | 0 | 0 | 0 |
| brain infarction & Metacognition &Neuropsychological Tests& Compute* | 0 | 0 | 0 | 0 | 0 | 0 |
| infarction & Metacognition &Neuropsychological Tests& Compute* | 0 | 0 | 0 | 0 | 0 | 0 |
| cerebral infarction & Metacognition &Neuropsychological Tests& Compute* | 0 | 0 | 0 | 0 | 0 | 0 |
| stroke & Cognition Disorders &Neuropsychological Tests& Compute* | 64 | 1 | 123 | 12 | 1 | 5 |
| cerebral hemorrhage & Cognition Disorders & Neuropsychological Testss& Compute* | 7 | 0 | 12 | 3 | 0 | 1 |
| hemorrhage & Cognition Disorders & Neuropsychological Tests& Compute* | 221 | 0 | 51 | 3 | 0 | 1 |
| brain infarction & Cognition Disorders &Neuropsychological Tests& Compute* | 9 | 0 | 27 | 8 | 0 | 0 |
| infarction & Cognition Disorders &Neuropsychological Tests& Compute* | 24 | 0 | 37 | 9 | 0 | 0 |
| cerebral infarction & Cognition Disorders &Neuropsychological Tests& Compute* | 6 | 0 | 15 | 6 | 0 | 0 |
| stroke & cognition & evaluation & Intelligen* | 14 | 41 | 29 | 2 | 1 | 2 |
| cerebral hemorrhage & cognition & evaluation & Intelligen* | 1 | 0 | 2 | 4 | 0 | 0 |
| hemorrhage & cognition & evaluation & Intelligen* | 81 | 8 | 13 | 4 | 0 | 0 |
| brain infarction & cognition & evaluation & Intelligen* | 5 | 10 | 12 | 2 | 0 | 0 |
| infarction & cognition & evaluation & Intelligen* | 8 | 15 | 18 | 2 | 0 | 0 |
| cerebral infarction & cognition & evaluation & Intelligen* | 5 | 2 | 5 | 2 | 0 | 0 |
| stroke & Metacognition & evaluation & Intelligen* | 1 | 1 | 1 | 0 | 0 | 0 |
| cerebral hemorrhage & Metacognition & evaluation & Intelligen* | 0 | 0 | 0 | 0 | 0 | 0 |
| hemorrhage & Metacognition & evaluation & Intelligen* | 1 | 0 | 0 | 0 | 0 | 0 |
| brain infarction & Metacognition & evaluation & Intelligen* | 0 | 0 | 0 | 0 | 0 | 0 |
| infarction & Metacognition & evaluation & Intelligen* | 0 | 0 | 0 | 0 | 0 | 0 |
| cerebral infarction & Metacognition & evaluation & Intelligen* | 0 | 0 | 0 | 0 | 0 | 0 |
| stroke & Cognition Disorders & evaluation & Intelligen* | 6 | 1 | 11 | 2 | 0 | 2 |
| cerebral hemorrhage & Cognition Disorders & evaluation & Intelligen* | 0 | 0 | 0 | 4 | 0 | 0 |
| hemorrhage & Cognition Disorders & evaluation & Intelligen* | 33 | 1 | 4 | 4 | 0 | 0 |
| brain infarction & Cognition Disorders & evaluation & Intelligen* | 2 | 0 | 3 | 2 | 0 | 0 |
| infarction & Cognition Disorders & evaluation & Intelligen* | 3 | 0 | 4 | 2 | 0 | 0 |
| cerebral infarction & Cognition Disorders & evaluation & Intelligen* | 2 | 0 | 1 | 2 | 0 | 0 |
| stroke & cognition & assessment & Intelligen* | 48 | 144 | 108 | 3 | 13 | 9 |
| cerebral hemorrhage & cognition & assessment & Intelligen* | 5 | 1 | 7 | 4 | 1 | 1 |
| hemorrhage & cognition &assessment & Intelligen* | 157 | 37 | 48 | 4 | 3 | 2 |
| brain infarction & cognition & assessment & Intelligen* | 11 | 25 | 33 | 2 | 6 | 0 |
| infarction & cognition & assessment & Intelligen* | 19 | 45 | 51 | 2 | 6 | 0 |
| cerebral infarction & cognition & assessment & Intelligen* | 9 | 3 | 8 | 2 | 3 | 0 |
| stroke & Metacognition & assessment & Intelligen* | 0 | 1 | 1 | 0 | 0 | 0 |
| cerebral hemorrhage & Metacognition & assessment & Intelligen* | 0 | 0 | 0 | 0 | 0 | 0 |
| hemorrhage & Metacognition & assessment & Intelligen* | 0 | 0 | 0 | 0 | 0 | 0 |
| brain infarction & Metacognition & assessment & Intelligen* | 0 | 1 | 0 | 0 | 0 | 0 |
| infarction & Metacognition & assessment & Intelligen* | 0 | 1 | 1 | 0 | 0 | 0 |
| cerebral infarction & Metacognition &assessment & Intelligen* | 0 | 0 | 1 | 0 | 0 | 0 |
| stroke & Cognition Disorders & assessment & Intelligen* | 32 | 1 | 43 | 3 | 5 | 8 |
| cerebral hemorrhage & Cognition Disorders & assessment & Intelligen* | 1 | 0 | 3 | 4 | 0 | 1 |
| hemorrhage & Cognition Disorders & assessment & Intelligen* | 55 | 0 | 11 | 4 | 0 | 2 |
| brain infarction & Cognition Disorders &assessment & Intelligen* | 8 | 0 | 9 | 2 | 0 | 0 |
| infarction & Cognition Disorders & assessment & Intelligen* | 10 | 0 | 14 | 2 | 0 | 0 |
| cerebral infarction & Cognition Disorders &assessment & Intelligen* | 5 | 0 | 5 | 2 | 0 | 0 |
| stroke & cognition &Mental Status and Dementia Tests & Intelligen* | 0 | 0 | 2 | 1 | 0 | 2 |
| cerebral hemorrhage & cognition &Mental Status and Dementia Tests & Intelligen* | 0 | 0 | 0 | 0 | 0 | 0 |
| hemorrhage & cognition &Mental Status and Dementia Tests& Intelligen* | 4 | 0 | 2 | 0 | 0 | 0 |
| brain infarction & cognition &Mental Status and Dementia Tests & Intelligen* | 0 | 0 | 0 | 0 | 0 | 0 |
| infarction & cognition &Mental Status and Dementia Tests& Intelligen* | 0 | 0 | 0 | 0 | 0 | 0 |
| cerebral infarction & cognition & Mental Status and Dementia Tests& Intelligen* | 0 | 0 | 0 | 0 | 0 | 0 |
| stroke & Metacognition &Mental Status and Dementia Tests& Intelligen* | 0 | 0 | 0 | 0 | 0 | 0 |
| cerebral hemorrhage & Metacognition &Mental Status and Dementia Tests& Intelligen* | 0 | 0 | 0 | 0 | 0 | 0 |
| hemorrhage & Metacognition &Mental Status and Dementia Tests& Intelligen* | 0 | 0 | 0 | 0 | 0 | 0 |
| brain infarction & Metacognition &Mental Status and Dementia Tests & Intelligen* | 0 | 0 | 0 | 0 | 0 | 0 |
| infarction & Metacognition &Mental Status and Dementia Tests& Intelligen* | 0 | 0 | 0 | 0 | 0 | 0 |
| cerebral infarction & Metacognition &Mental Status and Dementia Tests & Intelligen* | 0 | 0 | 0 | 0 | 0 | 0 |
| stroke & Cognition Disorders &Mental Status and Dementia Tests & Intelligen* | 0 | 0 | 1 | 1 | 0 | 1 |
| cerebral hemorrhage & Cognition Disorders & Mental Status and Dementia Tests& Intelligen* | 0 | 0 | 0 | 0 | 0 | 0 |
| hemorrhage & Cognition Disorders & Mental Status and Dementia Tests& Intelligen* | 2 | 0 | 0 | 0 | 0 | 0 |
| brain infarction & Cognition Disorders &Mental Status and Dementia Tests& Intelligen* | 0 | 0 | 0 | 0 | 0 | 0 |
| infarction & Cognition Disorders &Mental Status and Dementia Tests & Intelligen* | 0 | 0 | 0 | 0 | 0 | 0 |
| cerebral infarction & Cognition Disorders &Mental Status and Dementia Tests& Intelligen* | 0 | 0 | 0 | 0 | 0 | 0 |
| stroke & cognition &Neuropsychological Tests & Intelligen* | 43 | 19 | 96 | 2 | 2 | 9 |
| cerebral hemorrhage & cognition &Neuropsychological Tests & Intelligen* | 4 | 0 | 8 | 3 | 1 | 1 |
| hemorrhage & cognition &Neuropsychological Tests& Intelligen* | 102 | 6 | 38 | 3 | 1 | 2 |
| brain infarction & cognition &Neuropsychological Tests & Intelligen* | 9 | 5 | 32 | 2 | 1 | 0 |
| infarction & cognition &Neuropsychological Tests& Intelligen* | 14 | 8 | 41 | 2 | 1 | 0 |
| cerebral infarction & cognition & Neuropsychological Tests& Intelligen* | 8 | 0 | 10 | 2 | 1 | 0 |
| stroke & Metacognition &Neuropsychological Tests& Intelligen* | 1 | 0 | 1 | 0 | 0 | 0 |
| cerebral hemorrhage & Metacognition &Neuropsychological Tests& Intelligen* | 0 | 0 | 0 | 0 | 0 | 0 |
| hemorrhage & Metacognition &Neuropsychological Tests& Intelligen* | 1 | 0 | 0 | 0 | 0 | 0 |
| brain infarction & Metacognition &Neuropsychological Tests& Intelligen* | 0 | 0 | 0 | 0 | 0 | 0 |
| infarction & Metacognition &Neuropsychological Tests& Intelligen* | 0 | 0 | 0 | 0 | 0 | 0 |
| cerebral infarction & Metacognition &Neuropsychological Tests& Intelligen* | 0 | 0 | 0 | 0 | 0 | 0 |
| stroke & Cognition Disorders &Neuropsychological Tests& Intelligen* | 29 | 0 | 48 | 2 | 1 | 8 |
| cerebral hemorrhage & Cognition Disorders & Neuropsychological Testss& Intelligen* | 2 | 0 | 2 | 3 | 0 | 1 |
| hemorrhage & Cognition Disorders & Neuropsychological Tests& Intelligen* | 52 | 0 | 16 | 3 | 0 | 2 |
| brain infarction & Cognition Disorders &Neuropsychological Tests& Intelligen* | 7 | 0 | 9 | 2 | 0 | 0 |
| infarction & Cognition Disorders &Neuropsychological Tests& Intelligen* | 10 | 0 | 15 | 2 | 0 | 0 |
| cerebral infarction & Cognition Disorders &Neuropsychological Tests& Intelligen* | 5 | 0 | 5 | 2 | 0 | 0 |
|  | 3656 | 1268 | 2780 | 695 | 221 | 77 |

**Table S2.** Quality evaluation for randomized controlled studies (by modified Jadad scale)

| Included articles | Yun GJ, et al. ^[36]^ | Dalmaijer ES, et al. ^[40]^ |
| --- | --- | --- |
| Was the study described as randomized? | 1 | 1 |
| Was the approach of randomization appropriate? | 1 | 1 |
| Was the research described as blinding？ | 1 | 1 |
| Was the approach of blinding appropriate? | 1 | 1 |
| Was there a presentation of withdrawals and dropouts? | 0 | 0 |
| Was there a presentation of the inclusion/exclusion criteria? | 1 | 1 |
| Was the approach used to assess adverse effects described? | 0 | 0 |
| Was the approach of statistical analysis described? | 1 | 1 |
| Total scores | 6 | 6 |
| Comments | high quality | high quality |

**Table S3.** Quality evaluation for Case-control studies and Cohort studies (by NOS)

| Included articles | Study type | Selection | Comparability | Exposure | Total score | Comment |
| --- | --- | --- | --- | --- | --- | --- |
| Van Tuijl JH ^[46]^ | Case-control | 3 | 2 | 3 | 8 | A |
| Escartin G ^[49]^ | Case-control | 3 | 2 | 3 | 8 | A |
| Huang Y ^[19]^ | Prospective Cohort | 3 | 0 | 3 | 6 | B |
| Biffi A ^[20]^ | Prospective Cohort | 3 | 0 | 3 | 6 | B |
| Pendlebury ST ^[21]^ | Prospective Cohort | 2 | 0 | 3 | 5 | B |
| Jung HT ^[22]^ | Prospective Cohort | 1 | 0 | 2 | 3 | C |
| Toglia J ^[24]^ | Prospective Cohort | 3 | 0 | 2 | 5 | B |
| Sandel ME ^[25]^ | Prospective Cohort | 3 | 0 | 3 | 6 | B |
| Shopin L ^[26]^ | Prospective Cohort | 3 | 0 | 3 | 6 | B |
| Kliper E ^[27]^ | Prospective Cohort | 2 | 0 | 3 | 5 | B |
| Boussi-Gross R ^[28]^ | Prospective Cohort | 4 | 0 | 3 | 7 | A |
| Boussi-Gross R ^[29]^ | Retrospective Cohort | 2 | 0 | 2 | 4 | C |
| Cumming TB ^[33]^ | Prospective Cohort | 2 | 0 | 2 | 4 | C |
| Cumming TB ^[34]^ | Prospective Cohort | 2 | 0 | 3 | 5 | B |
| Kim BR ^[35]^ | Prospective Cohort | 3 | 2 | 2 | 7 | A |
| Ten Brink AF ^[38]^ | Retrospective Cohort | 2 | 0 | 2 | 4 | C |
| Wu YF ^[47]^ | Prospective Cohort | 3 | 2 | 3 | 8 | A |
| Fernández-Andújar M ^[48]^ | Prospective Cohort | 3 | 2 | 2 | 7 | A |
| Tung LC ^[50]^ | Prospective Cohort | 3 | 0 | 2 | 5 | B |
| Naidech AM ^[51]^ | Prospective Cohort | 3 | 0 | 3 | 6 | B |

Note: High quality = A; Moderate quality = B; Low quality = C.

**Table S4.** Quality evaluation for Cross-sectional studies (by AHRQ)

|  | Nir-Hadad SY ^[12]^ | Wall KJ ^[13]^ | Wall KJ ^[14]^ | Jovanovski D ^[15]^ | Carelli L ^[16]^ | Singh T ^[17]^ | Mostafavi SM ^[18]^ | Park HY ^[23]^ | Carlozzi NE ^[30]^ | Tulsky DS ^[31]^ | Nitsch KP ^[32]^ | Kim TW ^[37]^ | Ten Brink AF ^[39]^ | Schumacher R ^[41]^ | Deguchi K ^[42]^ | Yip CK ^[43]^ | Durisko C ^[44]^ | Schendel K ^[45]^ | Wallmark S ^[52]^ | Tippett WJ ^[53]^ |
| --- | --- | --- | --- | --- | --- | --- | --- | --- | --- | --- | --- | --- | --- | --- | --- | --- | --- | --- | --- | --- |
| (1)Define source of information (survey, record review) | ⊕ | ⊕ | ⊕ | ⊕ | ⊕ | ⊕ | ⊕ | ⊕ | ⊕ | ⊕ | ⊕ | ⊕ | ⊕ | ⊕ | ⊕ | ⊕ | ⊕ | ⊕ | ⊕ | ⊕ |
| (2)List inclusion and exclusion criteria for exposed and unexposed subjects (cases and controls) or refer to previous publications | ⊕ | ⊕ | ⊕ | ⊕ | ⊕ | ⊕ | ⊕ | ⊕ | ⊕ | ⊕ | ⊕ | ⊕ | ⊕ | ⊕ | ⊕ | ⊕ | - | ⊕ | ⊕ | ⊕ |
| (3)Indicate time period uesd for identifying patients | ⊕ | ⊕ | ⊕ | ⊕ | ⊕ | ⊕ | U | ⊕ | ⊕ | U | ⊕ | ⊕ | ⊕ | ⊕ | - | ⊕ | - | U | ⊕ | ⊕ |
| (4)Indicate whether or not subjects were consecutive if not population-based | U | - | U | - | U | U | U | U | U | U | U | U | U | - | ⊕ | U | U | U | U | U |
| (5)Indicate if evaluator of subjective components of study were masked to other aspects of the status of the participants | U | U | U | U | U | U | U | ⊕ | U | U | U | U | U | U | U | U | U | U | ⊕ | U |
| (6)Describe any assessments undertaken for quality assurance purposes | U | ⊕ | ⊕ | ⊕ | - | - | U | ⊕ | - | ⊕ | U | ⊕ | - | - | - | ⊕ | - | U | ⊕ | ⊕ |
| (7)Explain any patient exclusions from analysis | U | U | ⊕ | U | ⊕ | U | U | U | U | U | U | U | U | U | U | U | U | U | ⊕ | ⊕ |
| (8)Describe how confounding was assessed and /or controlled | ⊕ | ⊕ | ⊕ | ⊕ | ⊕ | ⊕ | ⊕ | U | ⊕ | ⊕ | ⊕ | U | ⊕ | ⊕ | U | U | U | ⊕ | U | ⊕ |
| (9)If applicable, explain how missing data were handled in the analysis | U | ⊕ | ⊕ | - | U | U | U | U | U | ⊕ | U | U | ⊕ | ⊕ | U | U | - | - | U | ⊕ |
| (10)Summarize patient response rates and completeness of data collection | ⊕ | ⊕ | ⊕ | ⊕ | ⊕ | ⊕ | ⊕ | ⊕ | ⊕ | ⊕ | ⊕ | ⊕ | ⊕ | ⊕ | ⊕ | ⊕ | ⊕ | ⊕ | ⊕ | ⊕ |
| (11)Clarify what follow-up, if any, was expected and the percentage of patients for which incomplete data or follow-up was obtained | NA | NA | NA | NA | NA | NA | NA | NA | NA | NA | NA | NA | NA | NA | NA | NA | NA | NA | NA | NA |
| Total score | 5 | 7 | 8 | 6 | 6 | 5 | 4 | 6 | 5 | 6 | 5 | 5 | 6 | 6 | 4 | 5 | 2 | 4 | 7 | 8 |
| Comments | B | B | A | B | B | B | B | B | B | B | B | B | B | B | B | B | C | B | B | A |

Note:Yes =⊕; No = - ; Unclear = U; Not applicable = NA; High quality = A; Moderate quality = B; Low quality = C.

**Supplementary Methods 1.** Modified Jadad scale for included randomized controlled trials

1) Was the study described as randomized?

a) yes (+1)

b) no (0)

2) Was the approach of randomization appropriate?

a) yes (+1)

b) no (-1)

C)not described (0)

3) Was the research described as blinding？^a^

a) yes (+1)

b) no (0)

4) Was the approach of blinding appropriate?

a) yes (+1)

b) no (-1)

C)not described (0)

5) Was there a presentation of withdrawals and dropouts?

a) yes (+1)

b) no (0)

6) Was there a presentation of the inclusion/exclusion criteria?

a) yes (+1)

b) no (0)

7) Was the approach used to assess adverse effects described?

a) yes (+1)

b) no (0)

8) Was the approach of statistical analysis described?

a) yes (+1)

b) no (0)

a: double-blind got 1 score, single-blind got 0.5 score.

**Supplementary Methods 2.** Newcastle-Ottawa Scale (NOS) for case-control studies

Note: A study can be awarded a maximum of one star for each numbered item within the Selection and Exposure categories. A maximum of two stars can be given for Comparability.

**Selection**

1) Is the case definition adequate?

a) yes, with independent validation (ex. diagnostic criteria, electromyography, neurologist) *

b) yes, via record linkage only (ex. identified through ICD codes on database records)

c) no description

2) Representativeness of the cases

a) consecutive or obviously representative series of cases *

b) potential for selection biases or not stated

3) Selection of Controls

a) community controls *

b) hospital controls

c) no description

4) Definition of Controls

a) no history of disease (endpoint) *

b) no description of source

**Comparability**

1) Comparability of cases and controls on the basis of the design or analysis

a) study controls for age *

b) study controls for gender/sex *

**Exposure**

1) Ascertainment of exposure

a) secure record (ex. field measurements, government databases, remote sensing) *

b) structured interview where blind to case/control status *

c) interview not blinded to case/control status

d) written self report or medical record only

e) no description

2) Same method of ascertainment for cases and controls

a) yes *

b) no

3) Non-Response rate (or missing data rate)

a) same rate for both groups *

b) rate different and non respondents described

c) rate different and/or no designation

**Supplementary Methods 3.** Newcastle-Ottawa quality assessment scale guide for cohort studies

Note: A study can be awarded a maximum of one star for each numbered item within the Selection and Outcome categories. A maximum of two stars can be given for Comparability

**Selection**

1) Representativeness of the exposed cohort

a) truly or somewhat representative of the average (exposed) in the community *

c) sampled from a special population (ex. company, hospital patients, insurance data)

d) no description of the derivation of the cohort

2) Selection of the non exposed cohort

a) drawn from the same community as the exposed cohort *

b) drawn from a different source

c) no description of the derivation of the non exposed cohort

3) Ascertainment of exposure

a) secure record (ex. field measurements, government databases, remote sensing) *

b) structured interview *

c) written self report

d) no description

4) Demonstration that outcome of interest was not present at start of study

a) yes *

b) no

**Comparability**

1) Comparability of cohorts on the basis of the design or analysis

a) study controls for age *

b) study controls for gender/sex *

**Outcome**

1) Assessment of outcome

a) independent or blind assessment or by reference to secure records (ex. medical files) *

b) record linkage (ex. identified through ICD codes on database records) *

c) self report (no reference to original medical records)

d) no description

2) Was follow-up long enough for outcomes to occur

a) yes (5+ years) *

b) no

3) Adequacy of follow up of cohorts

a) complete follow up - all subjects accounted for *

b) subjects lost to follow up unlikely to introduce bias (>= 80% follow up rate or description provided of those lost to follow up) *

c) follow up rate <80% and no description of those lost, or simply no statement

**Supplementary Methods 4.** the 11-item checklist which was recommended by Agency for Healthcare Research and Quality (AHRQ) for cross-sectional studies

1) Define source of information (survey, record review)

a) yes *

b) no

c)unclear

d)not applicable

2) List inclusion and exclusion criteria for exposed and unexposed subjects (cases and controls) or refer to previous publications

a) yes *

b) no

c)unclear

d)not applicable

3) Indicate time period uesd for identifying patients

a) yes *

b) no

c)unclear

d)not applicable

4) Indicate whether or not subjects were consecutive if not population-based

a) yes *

b) no

c)unclear

d)not applicable

5) Indicate if evaluator of subjective components of study were masked to other aspects of the status of the participants

a) yes *

b) no

c)unclear

d)not applicable

6) Describe any assessments undertaken for quality assurance purposes (e.g., test/retest of primary outcome measurements)

a) yes *

b) no

c)unclear

d)not applicable

7) Explain any patient exclusions from analysis

a) yes *

b) no

c)unclear

d)not applicable

8) Describe how confounding was assessed and /or controlled

a) yes *

b) no

c)unclear

d)not applicable

9) If applicable, explain how missing data were handled in the analysis

a) yes *

b) no

c)unclear

d)not applicable

10) Summarize patient response rates and completeness of data collection

a) yes *

b) no

c)unclear

d)not applicable

8) Clarify what follow-up, if any, was expected and the percentage of patients for which incomplete data or follow-up was obtained

a) yes *

b) no

c)unclear

d)not applicable
